# proBDNF-SorCS2 Axis Suppresses Osteogenesis and Augments Inflammation of Human Periodontal Ligament Stem Cells in Inflammatory Conditions

**DOI:** 10.1016/j.identj.2025.109294

**Published:** 2025-12-03

**Authors:** Lifei Pan, Yao Yang, Xiangbin Jia, Rui Zhao, Yuting Wang, Xiaoyue Guan, Tiezhou Hou

**Affiliations:** a Key Laboratory of Shaanxi Province for Craniofacial Precision Medicine Research, College of Stomatology, Xi’an Jiaotong University, Xi’an, Shaanxi, China; b Clinical Research Center of Shaanxi Province for Dental and Maxillofacial Diseases, College of Stomatology, Xian Jiaotong University, Xi’an, Shaanxi, China; c Department of Cariology and Endodontics, College of Stomatology, Xi’an Jiaotong University, Xi’an, Shaanxi, China; d Department of Cariology and Endodontics, Baoji Stomatological Hospital of Shaanxi, Baoji, China

**Keywords:** Periodontitis, ProBDNF, SorCS2, Periodontal ligament stem cells, Osteogenic differentiation

## Abstract

**Objectives:**

Periodontitis is a chronic inflammatory disease occurring in periodontal supporting tissues. Periodontal ligament stem cells (PDLSCs) are considered a promising source for periodontal regeneration and periodontitis treatment. Previous studies have shown that mature brain-derived neurotrophic factor (BDNF) can promote the multidirectional differentiation potential of PDLSCs. It has also been found that the proBDNF (precursor form of BDNF)–mediated signaling pathway is involved in immune-inflammatory diseases. In addition, some scholars have found that proBDNF and its receptor, sortilin-related central nervous system expressed receptor 2 (SorSC2), can induce cell apoptosis. However, the role of the proBDNF-SorCS2 axis in the development of periodontitis and its influence on human periodontal ligament stem cells (hPDLSCs) has not been elucidated. Our present experiment aims to investigate the relationship between the proBDNF-SorCS2 axis and periodontitis, as well as the role of proBDNF-SorCS2 in the osteogenic differentiation of hPDLSCs.

**Methods:**

In this study, 30 patients with periodontitis (PD) and 30 healthy controls (Cont) were selected to detect the protein expression level and the distribution of the proBDNF-SorCS2 axis in periodontal tissues. In addition, an inflammatory model of hPDLSCs was established to study the regulation of SorCS2 expression on inflammation and cell osteoblastic differentiation.

**Results:**

In our experimental results, we found that the protein expressions of proBDNF and receptor SorCS2 were significantly increased in periodontitis tissues and colocalized with the hPDLSC surface marker CD90. Importantly, we found that the reduction of SorCS2 in hPDLSCs inhibited the protein activity of the proBDNF-SorCS2 signaling axis, IL-6, and IL-1β, promoting osteogenic differentiation of hPDLSCs. We also note that the proBDNF-SorCS2 signaling axis is involved in the activation of the JNK signaling pathway.

**Conclusion:**

The protein levels of both proBDNF and its receptor SorCS2 were significantly upregulated in periodontitis tissues and involved in regulating the inflammatory expression level and osteogenic differentiation of hPDLSCs. The proBDNF-SorCS2 axis represents a promising target for novel, localized therapeutic interventions in periodontitis.

**Clinical relevance:**

The current study provides a new theoretical perspective and information for the treatment of periodontitis.

## Introduction

Periodontitis is a common oral inflammatory disease that causes the degradation of periodontal tissues, such as periodontal ligaments, alveolar bone, and cementum in infected teeth. Periodontitis is a chronic inflammatory disease driven by the interaction between an imbalanced periodontal microbiome and a dysregulated host immune response. The persistent challenge from this dysbiotic microbiota and their virulence factors leads to a hyperinflammatory state and the release of high levels of proinflammatory cytokines in periodontal tissues, resulting in the progressive destruction of the periodontal ligament and alveolar bone. Although significant research has been conducted on periodontitis, conventional periodontal treatments, such as scaling and root planning, are not always successful in preventing disease progression and achieving tissue regeneration, highlighting the need for more effective therapeutic strategies. Hence, a deeper understanding of periodontitis pathophysiology is critical for improving therapeutic outcomes.

The periodontal ligament stem cells (PDLSCs), which are the main source of mesenchymal stem cells (MSCs) in periodontal tissue, exhibit multipotent differentiation ability and possess osteogenic differentiation capacity.^1^ Aside from its aforementioned role, PDLSCs have also been shown to contribute to immunologic responses in periodontal regions.^2^ Recent studies have indicated that the suppression of inflammation and augmentation of the osteogenic ability of PDLSCs could facilitate the regeneration of periodontal tissues in the periodontitis course. In addition, Xiang et al^3^ discovered that inhibiting the inflammatory state of PDLSCs can accelerate the osteogenic differentiation of PDLSCs during the progression of periodontitis. Notably, our earlier study^4^ demonstrated that limiting the inflammatory state and enhancing the osteogenic capacity of PDLSCs can effectively suppress periodontitis progression and facilitate periodontal tissue regeneration. Therefore, in the pathogenesis of periodontal disease, it is extremely important to study whether specific genes play a role in enhancing the osteogenic ability of PDLSCs and inhibiting the inflammatory state.

Brain-derived neurotrophic factor (BDNF) is a neurotrophic factor that plays a crucial role in promoting the growth of neurons, developing the neuronal network, and enhancing the plasticity and function of synapses. Significantly, within neurons, BDNF can be secreted in the form of mature BDNF (mBDNF) as well as proBDNF. At present, research on the role of the BDNF gene in oral physiological and pathological conditions has focused primarily on mBDNF. Meng et al^5^ emphasized that upregulating mBDNF can enhance orthodontic tooth movement by reducing PDLSC senescence. Moreover, previous evidence^6^ suggests that elevating mBDNF can significantly strengthen the osteogenic potential of PDLSCs. Furthermore, recent studies have found that mBDNF can promote the proliferation of PDLSCs and their transformation into periodontal tissue cells.^7^ However, the precursor form of BDNF, proBDNF, has the opposite function of mBDNF. Specifically, in the central nervous system, proBDNF has a deleterious impact on neuronal remodeling, synaptic transmission, and synaptic plasticity in the hippocampus.^8^ In recent years, the role of proBDNF and its receptors in peripheral diseases has received extensive attention.^9^ There is evidence that proBDNF and its receptors are widely expressed in immune-mediated inflammatory diseases and involved in regulating the level of inflammation and apoptosis of related immune cells, thereby affecting the progression of the disease. However, it is not clear whether proBDNF and its receptors are also expressed in periodontitis and involved in regulating the inflammatory state and osteogenic differentiation ability of human PDLSCs (hPDLSCs) under inflammatory conditions.

Sortilin-related central nervous system expressed receptor 2 (SorCS2) is a member of the vacuolar protein sorting 10 protein (VPS10p) family, which are neuronal sorting receptors known for their involvement in the sorting of neurotrophins and their receptors in the central system.^10^ In a previous study, it was demonstrated that proBDNF’s biofunction is transmitted via p75NTR.^11^ However, in a recent study, SorCS2 has been identified as a newly discovered receptor for proBDNF. SorCS2 plays an essential role in regulating multiple signaling pathways that govern neuronal activity and function.^12^^,^^13^ The involvement of the proBDNF-SorCS2 axis in the pathogenesis of multiple diseases, including Alzheimer disease and Huntington disease, has been established.^14^ Notably, in our earlier study, it was found that Porphyromonas gingivalis lipopolysaccharide (Pg. LPS)-induced periodontitis stimulated high expression of the proBDNF-SorCS2 axis in the hippocampus, which was involved in depression-like behavior in mice induced by periodontitis. Moreover, in this experiment, we also demonstrated that the proBDNF-SorCS2 axis is expressed in periodontal tissues. However, the role of the proBDNF-SorCS2 axis in inflammation and the osteogenic potential of PDLSCs has not been clarified yet.

In this study, we evaluated the expression levels of proBDNF and SorCS2 in clinical samples and determined whether the biofunction of proBDNF was transduced via SorCS2 in lipopolysaccharide (LPS)–stimulated hPDLSCs. Furthermore, we also found a link between the proBDNF-SorCS2 axis and the JNK signaling pathway. Our findings could provide a novel foundation for understanding the pathogenesis of and developing therapies for periodontitis (PD).

## Methods

### Ethics

The collection of clinical and animal samples involved in this study was approved by the Medical Ethics Committee of Xi’an Jiaotong University Stomatological Hospital (Ethics No.: 2024-XJKQIEC-KY-QT-0036-002) and was carried out in strict accordance with the Ethical Principles of the Declaration of Helsinki. Before clinical samples were collected, all participants signed informed consent forms.

### Clinical sample collection

A total of 60 participants, consisting of 30 healthy individuals and 30 patients with chronic periodontitis, were recruited from the Hospital of Stomatology, Xi’an Jiaotong University. The involved participants were subsequently divided into two groups: the control group (Cont) and the chronic periodontitis group (PD).^15^ The inclusion and exclusion criteria were adopted according to the criteria employed in prior studies. Inclusion criteria: (1) aged between 18 and 65 years, (2) having at least 20 natural teeth (excluding third molars), (3) systemically healthy, and (4) provided informed consent. Exclusion criteria: (1) smoking; (2) history of using antibiotics, nonsteroidal anti-inflammatory drugs, steroids, immunosuppressants, β-blockers, calcium channel blockers, anticoagulants, or hormonal contraceptives within the 3 months prior to the study; (3) diagnosis of systemic diseases that may impair immune response (eg, diabetes, rheumatoid arthritis); (4) pregnancy or lactation; and (5) presence of orthodontic appliances. Participants in the healthy control group were defined by the absence of clinical inflammation, with <10% sites exhibiting bleeding on probing (BOP), no sites with probing pocket depth (PPD) ≥3 mm, and no radiographic evidence of bone loss. Patients in the periodontitis group were all diagnosed as having severe periodontitis by clinical examination by periodontal doctors and required at least 2 nonadjacent teeth with interdental clinical attachment loss (CAL) ≥5 mm and probing pocket depth (PPD) ≥6 mm, accompanied by radiographic bone loss extending to the mid-root or beyond. All clinical examinations were performed using a manual periodontal probe (William’s probe; Hu-Friedy), recording measurements at 6 sites per tooth (mesiobuccal, midbuccal, distobuccal, mesiolingual, midlingual, distolingual).

Healthy control tissue was taken from the upper one-third of the root of the premolars or the third molars that were extracted for orthodontic or preventive reasons. These areas have good clinical conditions and no signs of inflammation. Periodontitis tissue was collected during periodontal flap surgery from the periodontal pockets (depth ≥6 mm) or excised from the periodontal tissue of the tooth to be extracted. The collected tissue mainly consists of granulation tissue and inflamed connective tissue adjacent to the tooth root surface. After collection, the tissues were gently scraped on ice using a surgical blade, thoroughly rinsed with sterile phosphate-buffered saline (PBS), and stored in Eppendorf tubes. Parts of the tissues were fixed with 4% paraformaldehyde at room temperature for 48 hours, dehydrated, embedded with paraffin, and sliced to a thickness of 4 µm for histologic analysis, while others were immediately stored at –80 °C for Western blotting detection.

### Hematoxylin and eosin staining, immunohistochemical staining, and double immunofluorescent staining

The clinic samples were first dewaxed, rinsed, and dehydrated using a succession of graded ethanol. For hematoxylin and eosin (HE) staining, the specimens were stained using HE, following the instructions provided by the manufacturer (Solarbio, #G1120). The images were captured and examined using a light microscope (Nikon).

The process of immunohistochemistry (IHC) and immunofluorescent (IF) staining was carried out following the methodology reported in the prior study.^16^ Concisely, after being deparaffinized and rehydrated, the samples underwent treatment with a digestive solution to eliminate the antigen. Subsequently, to remove the endogenous peroxidase, the slices were incubated with 3% H_2_O_2_. The samples were then blocked with 5% bovine serum (BSA) to prevent nonspecific binding and then exposed to the appropriate primary antibodies, incubated at 4 °C. Following an 18-hour period, SABC-POD (mouse or rabbit IgG) kits (Boster, #SA1027 or SA1028) and DAB solution (Boster, #AR1027) were used for IHC. For IF, the slices were subjected to incubation with secondary antibodies, including anti-rabbit FITC (Boster, #BA1105) or antimouse CY3 antibodies (Boster, #BA1031). The nuclei were labeled with DAPI (Boster, #AR1176). IHC images were obtained using the Nikon Microscope Image System (Nikon), and immunofluorescence-positive stained cells were observed using a confocal fluorescence microscope (Olympus). The mean density of positive cells and the degree of colocalisation were assessed by ImageJ software (National Institutes of Health).

The primary antibodies used were anti-SorCS2 (Bioss, #bs-11963R), anti-proBDNF (Bioss, bs-4989R), anti-CD90 (Santa, #OX7), and anti–IL-1β (Bioss, #bs-0812R) (all antibodies were diluted 1:150).

### Isolation and culture of hPDLSCs

Intact healthy human premolars were extracted from 25 participants, aged 15 to 20 years, for orthodontic purposes. The extracted teeth were promptly preserved in Dulbecco’s modified Eagle’s medium (DMEM; Gibco). After being rinsed with sterile PBS for 3 to 5 times, periodontal ligament tissue in the middle third of the root surface was collected using a scalpel. Then, the harvested tissues were collected, finely chopped, and then grown in minimum essential α-modified Eagle medium (α-MEM; Gibco) supplemented with 20% fetal bovine serum (FBS; BI), 200 U/mL penicillin G, and 200 mg/mL streptomycin. The culture was maintained in a humidified atmosphere at 37 °C with 5% CO_2_. Once the PDLSCs reached a confluence of 80% to 90%, they were further cultured and then stored in liquid nitrogen for subsequent experiments. All PDLSCs used in the following study were at 2 to 5 passages.

### Cell-counting kit 8 assay

hPDLSCs at passages 2 to 4 were used for experiments. The cells were seeded into a 96-well tissue culture plate at a density of 3 × 10^3^ cells/well and then incubated at 37 °C for 24 hours. For the LPS, cells were treated with LPS (Sigma, #L2880) at concentrations of 0.1, 1.0, 10, or 50 µg/mL for 24, 48, or 72 hours at 37 °C. For anisomycin, cells were treated with anisomycin (MCE, #HY-18982) at a concentration of 0.025, 0.05, 0.1, 0.2, 0.5, 1.0, 2.0, 4.0, and 8.0 µM for 48 hours at 37 °C. After treatment, 10 µL of cell counting kit 8 (CCK-8) solution (Sparkjade, #CT0001) was added to each well, and the cells were incubated at 37 °C for 2 hours. Cell inhibition ratios were determined by reading the absorbance at 450 nm using a microplate reader.

### In vitro inflammation assay

To observe the impact of LPS (Sigma, #L2880) on the inflammatory state of hPDLSCs, the cells were placed in 6-well plates (2 × 10^5^ per well) and cultivated in a growth medium consisting of α-MEM supplemented with 10% FBS. Subsequently, the hPDLSCs were subjected to further stimulation with LPS at various concentrations, including 0, 0.1, 1, 10, and 50 μg/mL, at 37 °C for 24 hours. In addition, the hPDLSCs were further incubated with LPS at several time points: 0, 6, 12, 24, 48, and 72 hours, with a concentration of 1 µg/mL.

### Cell transfection

Lentiviral particles (GeneChem) were used to explore the regulatory capacity of SorCS2 in mediating the biofunction of proBDNF on periodontal inflammation. Three distinct short hairpin RNA (shRNA) sequences targeting SorCS2 were designed and synthesized by GeneChem. The sequences were as follows: shSorCS2-594, ACGCACAGAAGATCAGCT; shSorCS2-595, CTTTGAAGATCCTCAAGTT; and shSorCS2-596, CGAAGTATGCATTGCCAAA. The scrambled shRNA (TTCTCCGAACGTGTCACGT) served as the negative control. Knockdown efficiency was validated by Western blotting (Fig. 3B, 3C), and the most effective construct (shSorCS2-595) was chosen for all follow-up functional assays. hPDLSCs were seeded in 6-well plates at a density of 5 × 10^5^ cells per well, and then the cells were cultivated in a growth medium. Afterward, the medium was replaced with α-MEM supplemented with 10% FBS, 4% HitransG P, and lentivirus carrying a silencing cassette (shSorCS2) for 10 hours. hPDLSCs transfected with lentiviral particles served as controls. Expression of green fluorescent protein was observed by microscopy to evaluate the infection efficiency. Western blotting analysis was conducted to confirm the knockdown efficiency of SorCS2 in lentiviral particles transfected with hPDLSCs.

### Alkaline phosphatase staining and Alizarin red S staining

Alkaline phosphatase (ALP) staining and Alizarin red S (ARS) staining were conducted to investigate the effect of the proBDNF-SorCS2 axis on the regulation of the osteogenic differentiation of hPDLSCs under inflammatory conditions. hPDLSCs from passages 3 to 5 were cultured in either 24-well or 6-well plates. Then the cells were treated with an osteogenic induction medium, which consisted of 50 μM ascorbic acid 2 phosphate (Sigma-Aldrich, #49752), 10 μM β-glycerophosphate (Sigma-Aldrich, #G9422), and 100 nM dexamethasone (Sigma-Aldrich, #D4902) in α-MEM with 5% FBS and 1% antibiotic-antimycotic. The medium was replaced every 3 days. Following osteogenic induction for 7 and 21 days, hPDLSCs were rinsed with PBS and subsequently treated with 4% paraformaldehyde for 30 minutes. For ALP staining, the cells were treated with ALP staining solution (Beyotime, #P0321S) for 10 minutes at room temperature and then photographed by a digital camera and an Olympus FSX100 microscope for qualitative evaluation. The intensity of ALP staining was quantified using ImageJ software (National Institutes of Health). For ARS staining, hPDLSCs were treated with ARS solution (pH 4.2) (Beyotime, #C0138) for 1 hour, and the calcium-rich deposits made by hPDLSCs were stained dark red. Thereafter, cells were washed with PBS and photographed under an Olympus microscope with a digital camera. To get a qualitative measurement of the mineral clots, the cell sample was immersed in a solution of 10% acetic acid for 30 minutes, followed by heating at 85 °C for 10 minutes. Each well was treated with 10% ammonium hydroxide to neutralize the acid. Then, the solutions were gathered, and the optical density values were detected at a wavelength of 405 nm using a spectrophotometer.

### Western blotting and immunoprecipitation

Total proteins were extracted from human periodontal tissue and hPDLSCs using RIPA buffer (Beyotime, # P0013B) that contained 1% phenylmethanesulfonyl fluoride and 2% protease inhibitor.^17^ The protein concentration was subsequently determined by BCA assay (Beyotime, #P0010S). Then, 30 μg protein per sample was loaded onto a sodium dodecyl sulfate–polyacrylamide gel electrophoresis gel and subjected to electrophoresis at 100 V for 90 minutes. Subsequently, the protein samples were transferred to a polyvinylidene fluoride (PVDF) membrane (Millipore). The membranes were then blocked with a 5% milk solution for 1 hour at room temperature. Subsequently, the membranes were subjected to incubation with primary antibodies that specifically target the protein of interest at 4 °C overnight. Afterward, the PVDF membranes were incubated with a secondary antibody for 2 hours at room temperature. Finally, the relative quantification of protein expression levels was calculated using ImageJ software (National Institutes of Health). Primary antibodies included anti-proBDNF (1:500, #5H8; Santa), anti-SorCS2 (1:500, #A-10; Santa), anti–p-JNK (1:500, #G-7; Santa), anti-JNK (1:500, #D-2; Santa), and anti-GAPDH (1:1000, #bs-2188R; Bioss), anti–IL-1β (1:800, #bs-0812R; Bioss), anti–IL-6 (1:1000, #WL02841; WanLeibio), anti-ALP (1:500, #B-10; Santa), anti-OPN (1:500, #AKm2A1; Santa), anti-RUNX2 (1:500, #27-K; Santa), and anti-OCN (1:2000, #WLH4378; WanLeibio). For immunoprecipitation, an equal amount of total protein lysate was precleared using protein A + G Sepharose beads (Beyotime, #P2055). The supernatant was then incubated overnight at 4 °C and rotated in the presence of anti-SorCS2 antibodies (Bioss, #bs-11963R), after which the beads were added to the supernatant and rotated at 4 °C for 2 hours. Total lysate without primary antibody was used as an input control. The sheep IgG antibody was used as an isotype control. Immunoprecipitates were collected, and Western blotting was performed.

### Statistical analysis

The data were analysed using GraphPad Prism 9.0 (GraphPad Software). All data are presented as the mean ± standard deviation (SD). The normality of the data distribution was confirmed using the Shapiro-Wilk test. The homogeneity of variances was assessed using the Brown-Forsythe test. For comparisons between 2 groups, if the data passed both normality and variance homogeneity tests, an unpaired, 2-tailed Student *t* test was used. If the data passed the normality test but had unequal variances, an unpaired, 2-tailed Student *t* test with Welch’s correction was applied. For comparisons among multiple groups, if the data passed both normality and variance homogeneity tests, ordinary 1-way analysis of variance was performed, followed by Tukey’s post hoc test for multiple comparisons. If the data passed the normality test but had unequal variances, Welch’s corrected analysis of variance was performed, followed by Dunnett’s T3 test. All results were independently repeated 3 times, and *P* < .05 was considered statistically significant. *r* < 0.3 indicated no correlation between the 2 variables; 0.3 ≤ *r* ≤ 0.5, the 2 variables were in correlation; 0.5 ≤ *r* ≤ 0.7, the 2 variables were moderately correlated; and *r*>0.7, the 2 variables were highly correlated.

## Results

### proBDNF and SorCS2 are activated in human inflamed periodontal tissues

**Fig. 1:**
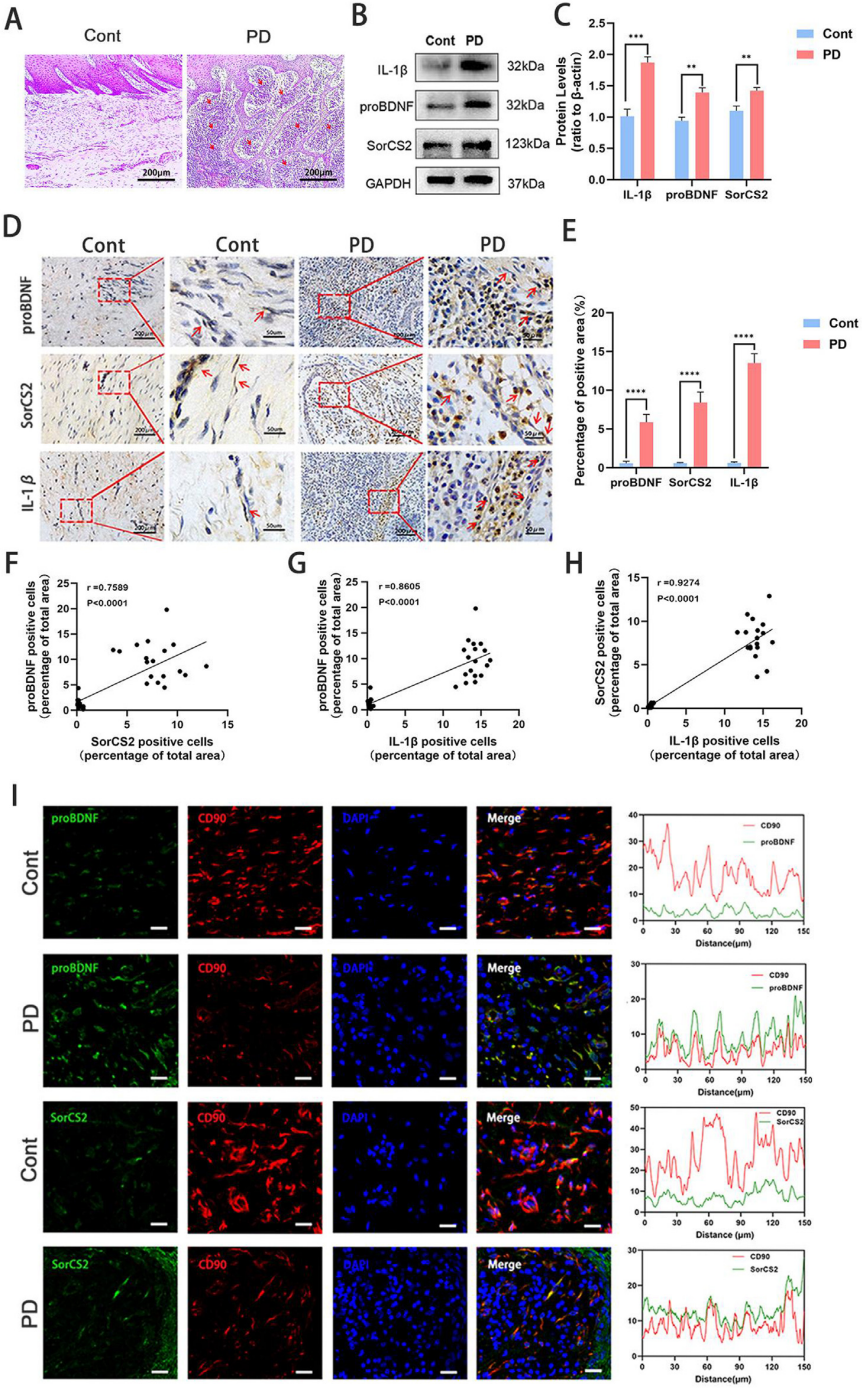
The expression of proBDNF, SorCS2, and IL-1β was increased in periodontitis. (A) HE staining analysis of periodontal tissue showing that the epithelial tissue in the periodontitis group expanded into the connective tissue and formed a mesh shape, with many inflammatory cells (red arrow) infiltrating around the epithelium. Scale bar: 200 μm. (B) Representative Western blotting image showing the expression levels of IL-1β, proBDNF, and SorCS2 in periodontal tissues, as mean ± SD. (C) Semi-quantification analysis of the indicated proteins based on Western blotting results. (D) Representative images of IHC staining for proBDNF, SorCS2, and IL-1β in the healthy periodontal tissues (Cont) and periodontitis tissues (PD). Scale bar: 200 μm (low magnification), 50 μm (high magnification). The distribution of proBDNF, SorCS2, and IL-1β in fibroblasts (red arrow). (E) Semi-quantification analysis of the indicated proteins based on IHC staining results. (F–H) Pearson correlation between proBDNF and SorCS2 (F), proBDNF and IL-1β (G), and SorCS2 and IL-1β (H). (I) (Left) Immunofluorescence staining of proBDNF/SorCS2 (green) and CD90 (red) in periodontal tissue of healthy participants (Cont) and patients with periodontitis (PD). The nucleus was identified by DAPI staining. Scale: 50 μm. (Right) Plots of pixel intensity of proBDNF/SorCS2 with CD90, with colors as in the merged images. *P < .05, **P < .01, ***P < .001, ****P < .0001; *r* ≥ 0.7 was considered to have a strong correlation between the 2 variables. All data, presented as mean ± SD, were analysed by unpaired 2-tailed Student *t* test (n = 3). BDNP, brain-derived neurotrophic factor; HE, hematoxylin and eosin; IHC, immunohistochemistry; IL, interleukin; SorCS2, sortilin-related central nervous system expressed receptor 2.

To clarify the role of the proBDNF-SorCS2 axis in periodontal tissues, samples of both healthy (Cont) and inflammatory periodontal tissues (PD) from humans were collected. Histologic analysis of periodontal tissues revealed substantial inflammatory cell infiltration and epithelial reticular hyperplasia in the periodontitis (PD) group, whereas healthy controls (Cont) showed no obvious inflammation and normal tissue architecture (Fig. 1A). At the molecular level, Western blot analysis demonstrated a significant upregulation of IL-1β, proBDNF, and SorCS2 in PD tissues compared to Cont tissues (Fig. 1B, 1C). Furthermore, IHC staining indicated that proBDNF, SorCS2, and IL-1β were mainly expressed in fibroblasts in healthy periodontal tissues, while in periodontal inflammatory tissues, proBDNF, SorCS2, and IL-1β were highly expressed in fibroblasts and inflammatory cells (Fig. 1D, 1E). To investigate the correlation of proBDNF, SorCS2, and IL-1β in healthy or pathological status of periodontal tissues, we conducted Pearson correlation analysis based on the ratio of immune-positive area to total area. The results indicated that a strong positive correlation was observed between the expression levels of proBDNF, SorCS2, and IL-1β in periodontal tissues (Fig. 1F-H). In addition, the colocalisation of proBDNF and SorC2 with CD90 (the biomarkers of stem cells) was validated in periodontal tissues, particularly in periodontitis samples, using IF staining (Fig. 1I). In summary, the above observations suggested that the proBDNF-SorCS2 axis may participate in the development of periodontitis and affect the biofunction of hPDLSCs.

### The expression of proBDNF-SorCS2 axis varies according to the inflammatory conditions in vitro

**Fig. 2:**
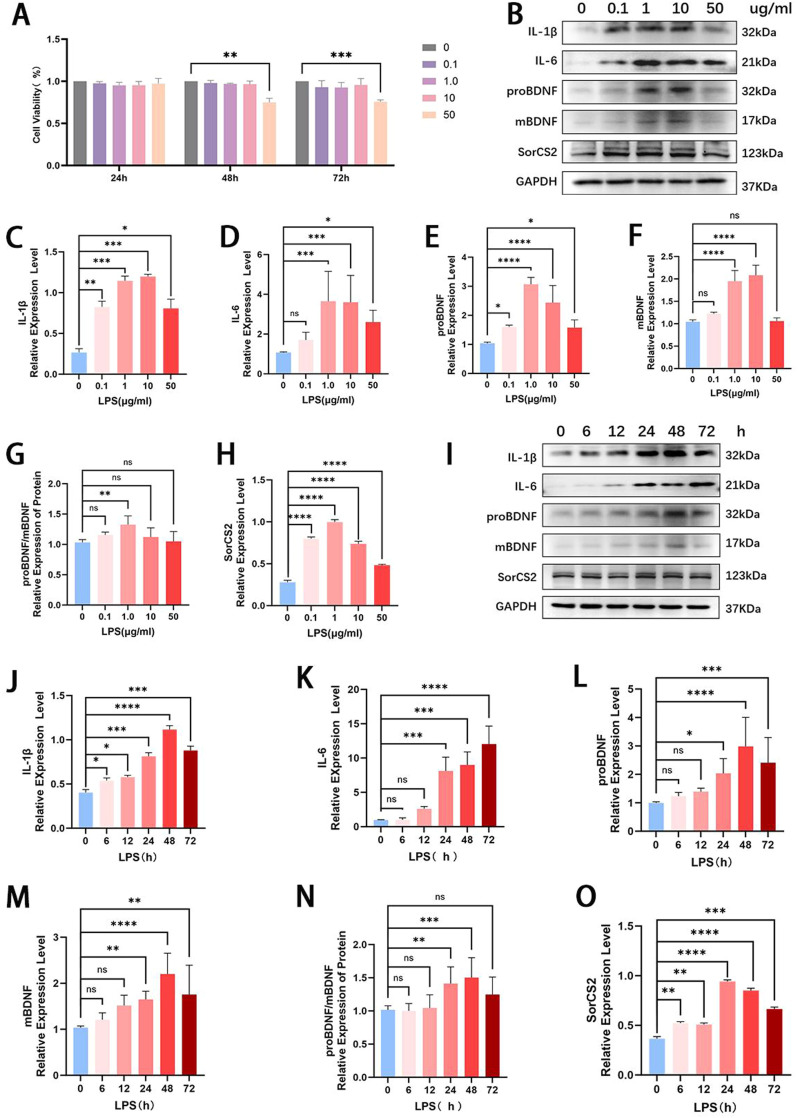
The expression of the proBDNF-SorCS2 axis in hPDLSCs in the inflammatory microenvironment. (A) The CCK-8 results show that LPS (0.1–10 µg/mL) did not influence the cell viability of hPDLSCs. (B) Representative Western blotting shows that different concentrations of LPS induced an inflammatory response in hPDLSCs, and the expression levels of IL-1β, IL-6, proBDNF, mBDNF, and SorCS2 were detected. (C-H) Quantitative analysis of protein expression levels at different concentrations of LPS. (I) Representative Western blotting shows that LPS induced the inflammatory response of hPDLSCs at different times, and the expression levels of IL-1β, IL-6, proBDNF, mBDNF, and SorCS2 were detected. (J-O) Quantitative analysis of protein expression levels of 1 μg/mL LPS under different treatment times. *P < .05, **P < .01, ***P < .001, ****P < .0001; ns., *P* > .05. All data, presented as mean ± SD, were analysed by 1-way analysis of variance (n = 3). BDNP, brain-derived neurotrophic factor; CCK-8, cell counting kit 8; hPDLSC, human periodontal ligament stem cell; IL, interleukin; LPS, lipopolysaccharide; mBDNP, mature brain-derived neurotrophic factor; SorCS2, sortilin-related central nervous system expressed receptor 2.

To model periodontitis in vitro, hPDLSCs were treated with LPS. CCK-8 assays confirmed that LPS concentrations up to 10 μg/mL did not significantly affect cell viability over 72 hours, whereas 50 μg/mL reduced viability after 48 hours. Therefore, subsequent experiments used concentrations ≤10 μg/mL (Fig. 2A). To validate the inflammatory model, IL-1β and IL-6 expression were measured by Western blot analysis. The findings revealed that LPS stimulation significantly augmented IL-1β and IL-6 expression compared to the untreated. Specifically, the expression of IL-1β and IL-6 both rose with increasing LPS concentration from 1.0 to 10 μg/mL but decreased slightly at 50 μg (Fig. 2B-D). Moreover, in the hPDLSCs treated with 1μg/mL LPS, we found that the expression of IL-1β increased over time compared to the control group. IL-1β expression increased from 6 to 48 hours and thereafter dropped after 72 hours (Fig. 2I, 2J). However, the expression level of IL-6 began to increase after 24 hours (Fig. 2I, 2K).

Once the inflammatory state of the cell model was verified, the expression of proBDNF and SorCS2 was further assessed. The results demonstrated that the expression levels of proBDNF correspond to the expression of IL-1β (Fig. 2B, 2E, 2I, 2L). The results showed that when treated with LPS at a concentration of 1.0 μg/mL for 24, 48, or 72 hours, the expression of mBDNF significantly increased, while the ratio of proBDNF/mBDNF was significantly higher than that of the control group at 48 hours (*P* < 0.001; Fig. 2B, 2F, 2G, 2I, 2M, 2N). Regarding the expression of SorCS2, we observed a considerable increase in its expression following the administration of LPS. In detail, the expression of SorCS2 enhanced when the concentration of LPS increased from 0.1 to 1μg/mL but showed a modest reduction at 10 and 50 μg/mL (Fig. 2B, 2H). In addition, in the hPDLSCs treated with 1 μg/mL LPS, we observed a progressive rise in the expression of SorCS2 over time, compared to the control group. The expression of SorCS2 was upregulated from 6 to 24 hours and thereafter decreased at 48 and 72 hours (Fig. 2I, 2O). As shown above, it is suggested that proBDNF-SorCS2 may be involved in the pathogenesis of periodontitis by augmenting the inflammatory status in PDLSCs.

### Silencing SorCS2 alleviates the inflammatory responses induced by LPS

**Fig. 3:**
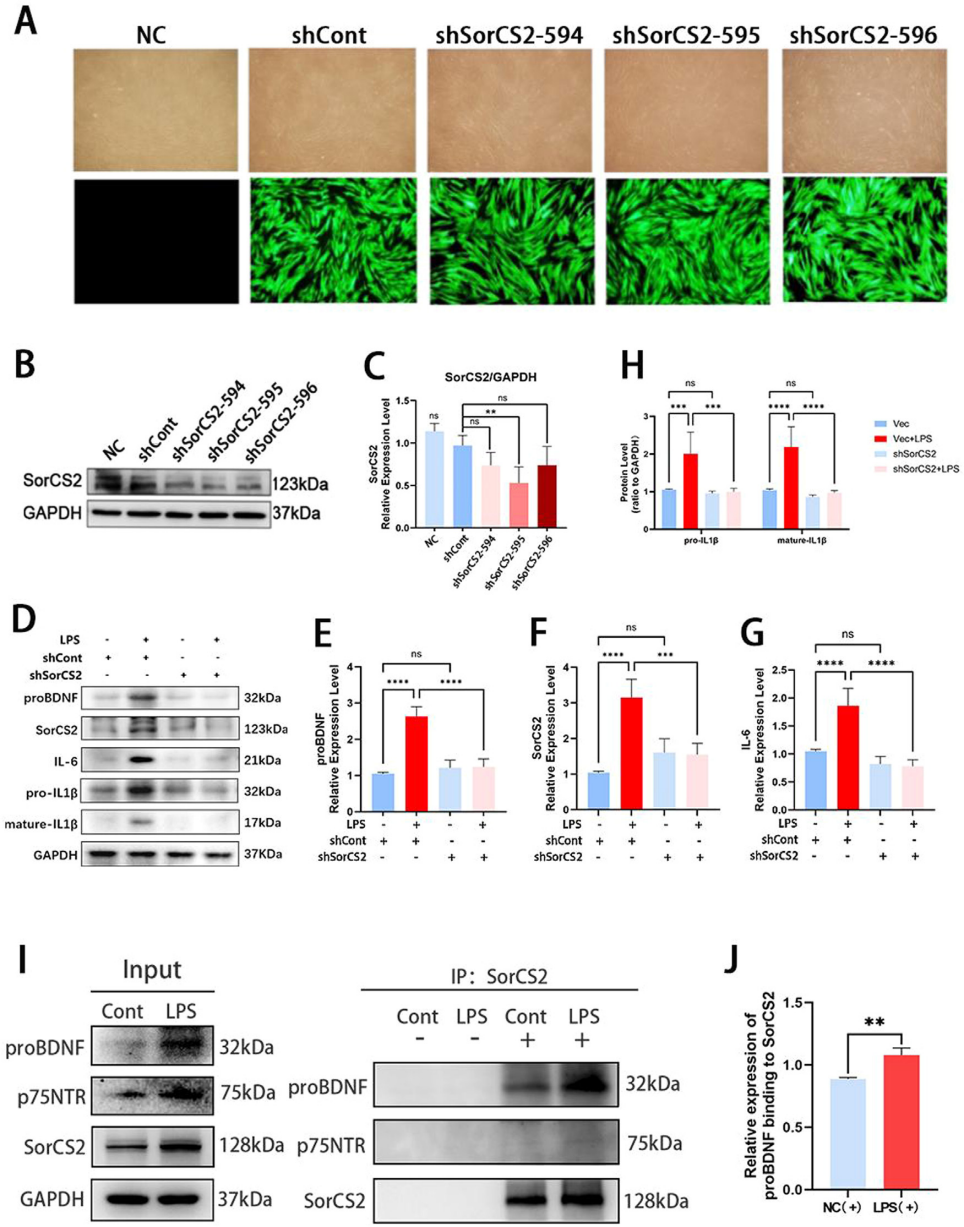
Silencing SorCS2 could decrease the expression level of the proBDNF-SorCS2 signaling axis and inflammatory cytokines in infected hPDLSCs. (A) Representative fluorescent images were used to observe the effectiveness of lentivirus transfection. (B, C) The efficiency of SorCS2 knockdown was detected by Western blotting. shSorCS2-595 was selected for all subsequent functional experiments due to its superior knockdown efficiency. (D) Representative images of protein expression levels of the proBDNF-SorCS2 signaling axis, IL-1β and IL-6 in shCont groups, and shSorCS2 groups under LPS stimulation. (E–H) The quantification of each protein expression. Following the silencing of SorCS2, the expression levels of the proBDNF-SorCS2 axis, as well as the proteins IL-6 and IL-1β, were reduced. (I–J) Endogenous interaction of SorCS2 with proBDNF and p75NTR. No anti-immunoprecipitation was used as a negative control. LPS, inflammation group; NC, control group; (+), immunoprecipitation with anti-SorCS2 antibody; (–), immunoprecipitation with no primary antibody (H). The quantitative analysis of co-IP (I). **P < .01, ***P < .001, ****P < .0001; ns., *P* > .05. All data are presented as mean ± SD. (E–H) were analysed by 1-way analysis of variance, and an unpaired 2-tailed Student *t* test for (J) (n = 3). BDNP, brain-derived neurotrophic factor; hPDLSC, human periodontal ligament stem cell; IL, interleukin; LPS, lipopolysaccharide; SorCS2, sortilin-related central nervous system expressed receptor 2.

To observe the functional role of SorCS2, we established a stable SorCS2 gene knockout model in hPDLSCs using lentiviral shRNA (shSorCS2-594/595/596). The transfection efficiency was confirmed by Western blotting and fluorescence imaging experiments, and transfection with the shSorCS2 lentivirus at 595 could effectively reduce the content of SorCS2 protein (Fig. 3A-C). Thus, in subsequent explorations, we will use 595 to treat the cells. Afterward, hPDLSCs were subjected to LPS treatment. The results showed that compared to the shCont group, the expression of proBDNF, SorCS2, IL-1β, and IL-6 was markedly increased in the shCont + LPS group. However, inhibition of SorCS2 protein level in PDLSCs under inflammatory conditions could decrease the expression of IL-1β and IL-6 (Fig. 3D, 3G, 3H). Importantly, we found that silencing SorCS2 can inhibit the expression of proBDNF (Fig. 3D, 3E), and we speculated that the reason might be that the inhibition of SorCS2 could affect the expression of its coreceptors. Moreover, we detected the expression of p75NTR, a possible coreceptor of the proBDNF-SorCS2 axis, via co-immunoprecipitation to observe whether p75NTR participated in the regulation of the proBDNF-SorCS2 axis by SorCS2 in hPDLSCs. However, it was found that there was no significant change in p75NTR compared with the control group (Fig. 3I, 3J).

### Silencing SorCS2 could promote osteogenic differentiation of hPDLSCs in the inflammatory conditions

**Fig. 4:**
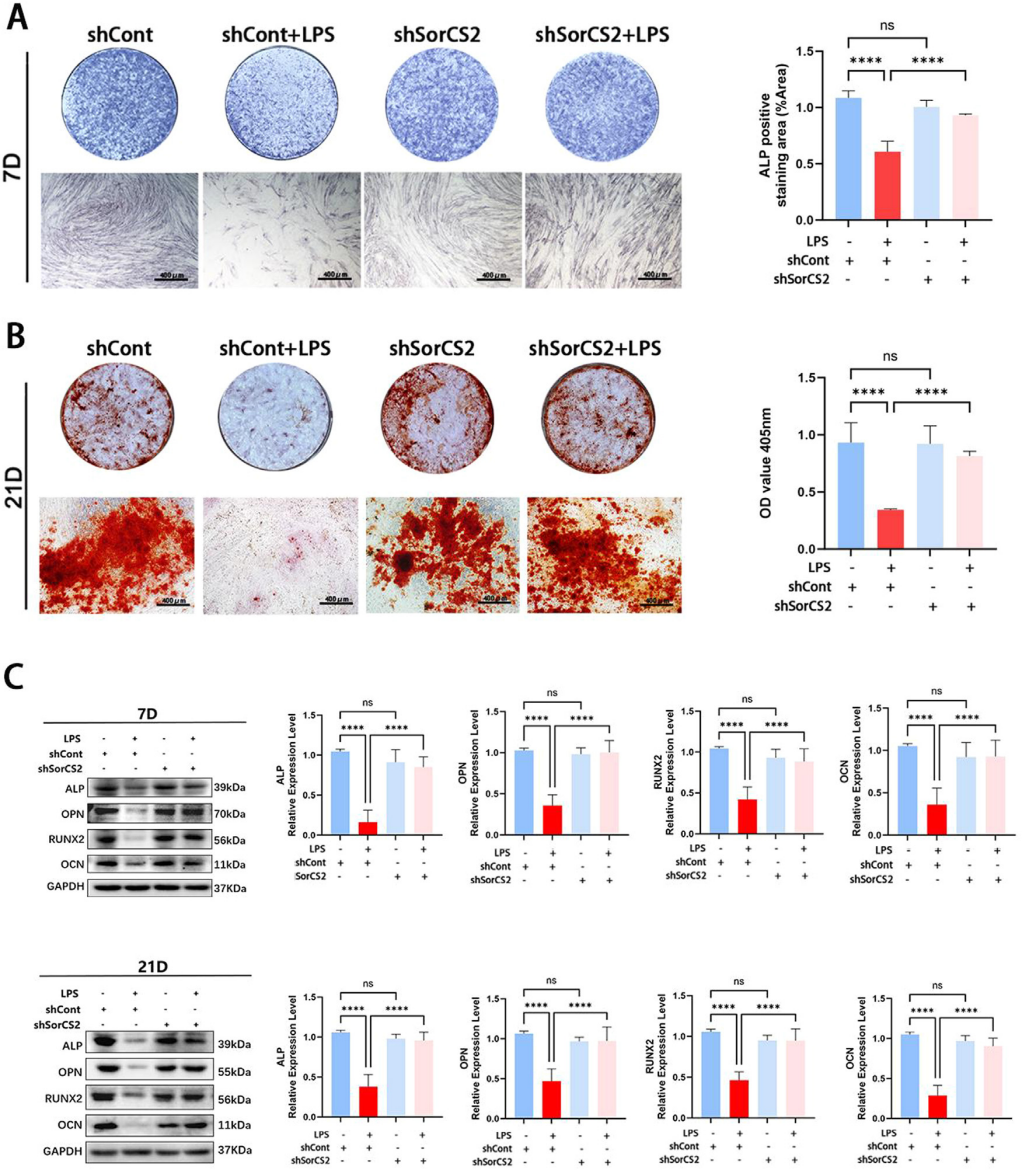
The effect of SorCS2 on osteogenic differentiation in hPDLSCs. (A) ALP activity was measured at 7 days after osteogenesis induction, with the quantification of the ALP staining results. (B) ARS results were performed at 21 days after osteogenic induction, with the quantification of the ARS staining results. (C) The protein expression levels of ALP, OPN, RUNX2, and OCN were detected via Western blotting at 7 and 21 days after osteogenic induction, with the quantitative analysis of the protein expression of ALP, OPN, RUNX2, and OCN. ****P < .0001, ns., *P* > .05. All data, presented as mean ± SD, were analysed by 1-way analysis of variance (n = 3). ALP, alkaline phosphatase; ARS, Alizarin red staining; hPDLSC, human periodontal ligament stem cell; SorCS2, sortilin-related central nervous system expressed receptor 2.

PDLSCs can differentiate into osteoblasts, which is beneficial to periodontal bone regeneration and mineralisation, as well as bone repair as periodontitis progress.^18^ However, studies have found that when periodontitis occurs, the inflammatory microenvironment could significantly inhibit the osteogenic differentiation ability of PDLSCs. In our previous results, we have proved that the proBDNF-SorCS2 axis is positively correlated with the occurrence and development of periodontitis, as observed in clinical samples and cellular inflammation models, and SorCS2 could affect the expression of the proBDNF-SorCS2 signaling axis in inflammatory environments. Furthermore, we studied the effect of the proBDNF-SorCS2 signal axis on osteogenic differentiation of hPDLSCs under inflammatory conditions.^18^ The cells were first infected with shSorCS2 lentivirus and then exposed to LPS at 10 μg/mL.^19^ Afterward, ALP and ARS staining were used to evaluate the osteogenic differentiation of hPDLSCs. The results showed that the inflammatory microenvironment significantly reduced the intensity of ALP staining and the number of red mineralized nodules. The osteogenic ability of the Vector + LPS group was significantly reduced compared with the Vector group. In contrast, SorCS2 knockdown improved the early and late osteogenic capacity of hPDLSCs in an inflammatory environment (Fig. 4A, 4B). In order to further detect the expression levels of osteoblast protein, the expressions of ALP, OPN, RUNX2, and OCN were detected by Western blotting on days 7 and 21. The results showed that knocking down the expression of SorCS2 could improve the inhibitory effect of the inflammatory environment on the osteoblastic ability of hPDLSCs (Fig. 4C). In conclusion, the above findings suggest that blocking the proBDNF-SorCS2 axis could enhance the osteogenesis ability of hPDLSCs under inflammatory conditions.

### proBDNF-SorCS2 axis regulates the inflammatory states and osteogenic differentiation potential of PDLSCs under inflamed circumstances via the JNK signaling pathway

**Fig. 5:**
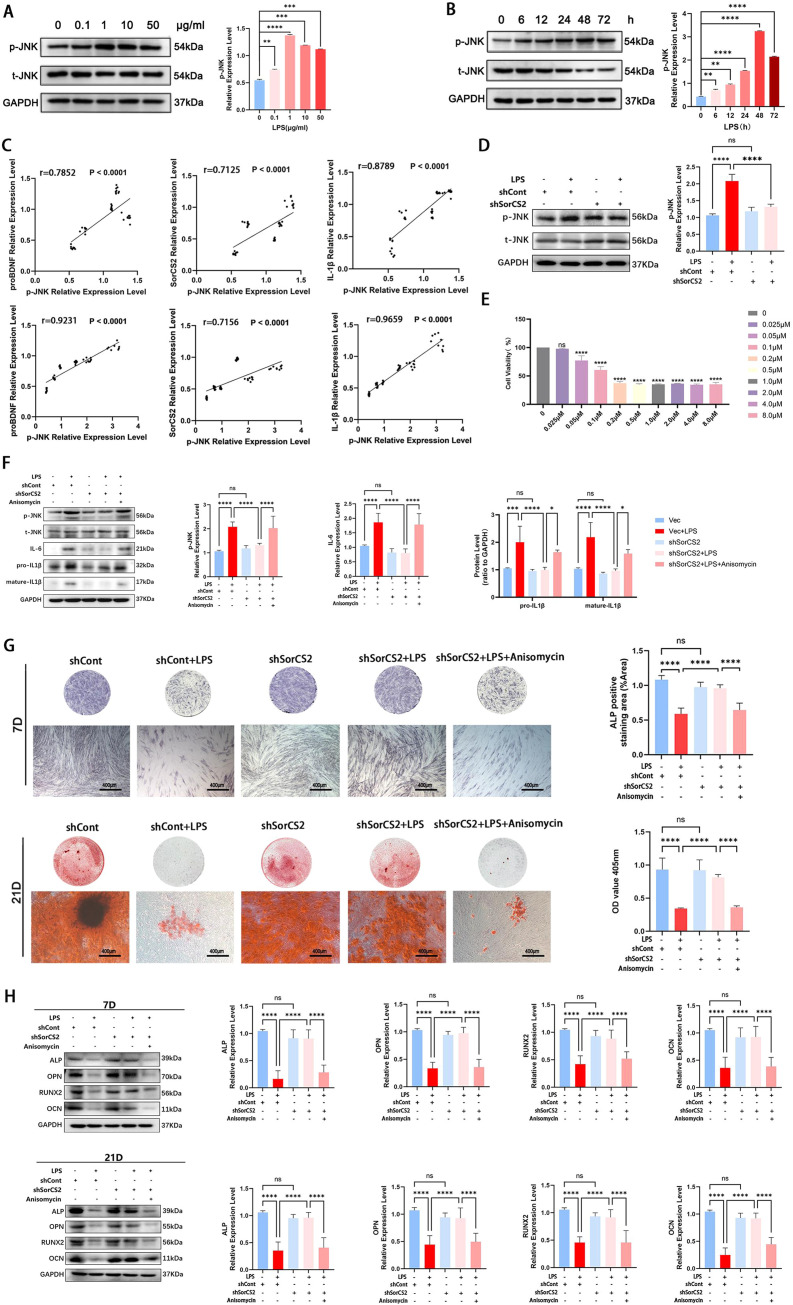
The JNK signaling pathway is involved in the regulation of PDLSCs by the proBDNF-SorCS2 axis under the inflammatory microenvironment. (A) Representative image of JNK signaling pathway expression levels in hPDLSCs stimulated by LPS at different concentrations, along with the quantitative analysis of the protein expression of the JNK signaling pathway with different LPS concentrations. (B) Representative image of JNK signaling pathway expression levels in hPDLSCs stimulated by LPS at different times, as well as the quantitative analysis of the protein expression of the JNK signaling pathway with different LPS working hours. (C) Pearson correlation between p-JNK/JNK and proBDNF, SorCS2 or IL-1β in hPDLSCs. There was a positive correlation between proBDNF and pJNK/JNK, SorCS2 and pJNK/JNK, and IL-1β and pJNK/JNK with different LPS concentrations, as well as a positive correlation between proBDNF and pJNK/JNK, SorCS2 and pJNK/JNK, and IL-1β and pJNK/JNK with different LPS working hours. (D) Representative images of protein expression levels of JNK signaling pathways in viral control groups and transfected cells under LPS stimulation, with quantitative expression of the JNK signaling pathway in different groups. (E) The CCK-8 assay results showed that at a concentration of 0.025 μM, anisomycin had no effect on the cell viability of hPDLSCs. (F) Enhancement of JNK signaling by anisomycin inhibited the anti-inflammatory effect of knocking down SorCS2 under proinflammatory conditions. Representative images of protein expression levels for the JNK pathway, IL-1β, and IL-6, with the quantification of each protein expression. (G) ALP activity was measured at 7 days after osteogenesis induction, with the quantification of the ALP staining results. ARS results were performed 21 days after osteogenic induction, with the quantification of the ARS staining results. (H) The protein expression levels of ALP, OPN, RUNX2, and OCN were detected via Western blotting at 7 and 21 days after osteogenic induction, with the quantitative analysis of the protein expression of ALP, OPN, RUNX2, and OCN. *P < .05, **P < .01, ***P < .001, ****P < .0001; *r* ≥ 0.7 was considered to have a strong correlation between the 2 variables. All data, presented as mean ± SD, were analysed by 1-way analysis of variance (n = 3). BDNP, brain-derived neurotrophic factor; CCK-8, cell counting kit 8; hPDLSC, human periodontal ligament stem cell; IL, interleukin; LPS, lipopolysaccharide; PDLSC, periodontal ligament stem cell; SorCS2, sortilin-related central nervous system expressed receptor 2.

The JNK signaling pathway, an important pathway involved in osteogenic differentiation and inflammatory response of PDLSCs,^20^^,^^21^ is involved in the progression of multiple diseases that are regulated by proBDNF.^22^^,^^23^ Thus, in this study, we investigated whether the JNK signaling pathway was involved in the regulatory effect of the proBDNF-SorCS2 axis on PDLSCs under inflammatory conditions. First, we detected the expression of JNK and phosphorylated-JNK (p-JNK) in LPS-infected PDLSCs. The results showed that the JNK signaling pathway in hPDLSCs was activated after LPS treatment, and the expression level reached the peak at a concentration of 1 μg/mL for 48 hours and then decreased, but the expression level of p-JNK/JNK was still significantly increased compared with the non-LPS treatment group (Fig. 5A, 5B). Then we analysed the correlation between the expression of proBDNF and p-JNK/JNK, SorCS2 and p-JNK/JNK, and IL-1β and p-JNK/JNK. The results showed that there is a strong positive correlation between proBDNF and p-JNK/JNK, SorCS2 and p-JNK/JNK, and IL-1β and p-JNK/JNK (Fig. 5C). Moreover, by suppressing the expression of SorCS2 in LPS-infected PDLSCs, we further observed that the ratio of p-JNK to JNK decreased significantly (Fig. 5D). The above data implied that the JNK signaling pathway participates in the regulatory effect of the proBDNF-SorCS2 signaling axis in PDLSCs under inflammatory conditions. To further confirm these observations, we employed the JNK pathway activator (anisomycin) in SorCS2-knockdown hPDLSCs under LPS treatment. CCK-8 results showed that the treatment of cells with anisomycin at a concentration of 0.025 μM for 48 hours had no significant effect on cell viability (Fig. 5E). After applying anisomycin, the results showed that the activation of the JNK signal pathway successfully reversed the beneficial effects of SorCS2 silencing, leading to a reincrease in inflammation and a resuppression of osteogenesis (Figure 5F-H).

## Discussion

Our study found a previously unrecognized role for the proBDNF-SorCS2 axis as a critical negative regulator of periodontal homeostasis.^5^, ^6^, ^7^ While the neurotrophic functions of mature BDNF in oral biology are well documented, its precursor, proBDNF, has remained largely unexplored in periodontal disease. The present work demonstrates that the proBDNF-SorCS2 axis is significantly upregulated in human periodontal disease tissues, and it plays a major role as an inflammatory driver and a potent osteogenic inhibitor in hPDLSCs. Therefore, inhibition of proBDNF-SorCS2 signaling may provide a potential target for localized intervention for regulating the immune-inflammatory response and periodontal tissue reconstruction in patients with periodontitis.

Although the inflammatory cytokines involved in periodontitis are known and can be effectively alleviated by current periodontal treatment strategies, the ultimate goal of effective periodontal tissue regeneration remains difficult to achieve.^24^ The PDLSCs have been shown to be ideal cells for periodontal tissue engineering. However, the osteogenic differentiation ability of these cells is weakened in periodontitis, making it unfavorable for periodontal tissue repair. Therefore, maintaining the activity of PDLSCs in the inflammatory microenvironment and promoting the osteogenic differentiation of PDLSCs may be critical for better periodontitis treatment outcomes. This study shows that inflammatory signaling pathways and inflammatory molecules are related to the pathogenesis of periodontitis, and blocking the expression of these pathways can reduce the level of inflammation in the inflammatory microenvironment and improve the possibility of osteogenic differentiation of cells.

### proBDNF-SorCS2 is upregulated in the periodontal tissues of patients with periodontitis

The proBDNF-mediated signaling pathway is involved in many diseases related to immune inflammation.^25^^,^^26^ Therefore, understanding the expression and function of neurotrophic factors in periodontal tissues of patients with periodontitis may increase the evaluation of the pathogenesis of periodontitis and the development of new treatment strategies. In this study, we investigated the regulatory role of proBDNF-SorCS2, a signaling pathway mediated by proBDNF, in the progression of inflammatory disease in patients with periodontitis. In our present results, compared with healthy participants, the protein levels of proBDNF-SorCS2 and inflammatory factors in the periodontal tissues of patients with periodontitis were significantly increased, indicating that the high expression of proBDNF-SorCS2 signal transduction in periodontal tissues is related to periodontitis when immune inflammation occurs. It may play an important role in periodontitis progression and periodontal tissue healing.

### Expression pattern of proBDNF-SorCS2 during periodontitis progression

PDLSCs play an important role in maintaining periodontal homeostasis.^27^ The normal periodontal environment is in a state of dynamic balance between cell proliferation and apoptosis, and the invasion of periodontal pathogens will impair the self-renewal function of PDLSCs.^9^ BDNF is a neurotrophic factor gene that is widely expressed in the central nervous system and is basically synthesized as a precursor (proBDNF), which is then cleaved to mBDNF by proteases.^28^ Studies have shown that dental MSCs, which mainly include apical papilla (SCAP), dental pulp stem cells (DPSCs), and PDLSCs, express BDNF and have a significant effect on nerve growth. Compared with BDNF, proBDNF can play a positive role in promoting apoptosis, cytokine processing, and the inflammatory response. The results of this study show that compared to the control group, PDLSCs in an inflammatory state significantly expressed proBDNF, mBDNF, and SorCS2, indicating that the BDNF gene overall was upregulated during the inflammatory response, and its receptors were activated. At the LPS concentration of 1 μg/mL for 48 hours, the ratio of proBDNF/mBDNF significantly increased. The increase in the ratio indicates a shift toward proinflammatory signals within cells. The synthesis of the BDNF precursor form within the cells has increased, or the process of proBDNF converting to mBDNF has been inhibited. The specific mechanism of the upregulation of proBDNF expression and the changes in the ratio of proBDNF/mBDNF are worthy of further exploration regarding their role in the progression of periodontitis. Based on the results, we preliminarily conclude that when periodontal disease occurs, the expression of proBDNF-SorCS2 in the local tissues of the lesion area is higher, and the expression of proBDNF-SorCS2 in the PDLSCs of the lesion area also significantly increases. During the progression of periodontal disease, proBDNF-SorCS2 may exert a proinflammatory effect on PDLSCs.

### shSorCS2 pretreatment decreased the expression levels of proBDNF-SorCS2 and inflammation and improved the osteogenic differentiation ability of PDLSCs

In this study, lentiviral transfection of PDLSCs was performed to inhibit SorCS2 expression. SorCS2 knockdown alone (shSorCS2 group) did not significantly alter the expression of inflammatory factors or osteogenic proteins compared to the control (shCont group), indicating that SorCS2 deficiency does not disrupt basal cellular physiology. However, in the inflammatory environment induced by LPS, compared with the shCont + LPS group, the expression of inflammatory factors in the shSorCS2 + LPS group was significantly reduced, while the osteogenic indicators significantly increased. These results collectively confirm that the regulatory effect of inhibiting SorCS2 on inflammation and osteogenic differentiation depends on the LPS-induced inflammatory environment, that is, this specificity enhances its therapeutic potential, suggesting that targeting SorCS2 may modulate pathologic processes without disrupting physiologic tissue repair. Notably, our results show that inhibition of SorCS2 reduces the expression of proBDNF and proinflammatory cytokines in cells.^29^ We suspect that the reduced expression level of proBDNF is related to the common coreceptor p75NTR of the proBDNF-SorCS2 axis. Therefore, we also examined whether p75NTR participated in the proBDNF-SorCS2 axis-mediated inflammatory response in hPDLSCs. However, no significant binding effect of p75NTR with SorCS2 was found in our results.^11^ Previous studies have found that SorCS2, as a key receptor of proBDNF, mediates the internalisation of proBDNF through specific binding under pathological conditions. This process can activate downstream signaling pathways and play cytologic functions of promoting apoptosis and growth cone collapse.^30^ At the same time, this process can also avoid protease degradation of proBDNF by reducing the residence time of ligands on the cell surface. When SorCS2 is inhibited, the internalisation of proBDNF is blocked due to the loss of ability to bind to the receptor. Long-term exposure of proBDNF to the surface of cell membranes may lead to structural instability, significantly increasing the probability of cutting by plasmin or matrix metalloproteinases (MMPs), resulting in rapid degradation of unbound proBDNF. In conclusion, the decrease of proBDNF after SorCS2 knockdown may be the result of surface retention degradation and loss of stability.

It was found that the osteogenic differentiation ability of PDLSCs was significantly weakened in the inflammatory microenvironment induced by LPS or tumor necrosis factor α (TNF-α), resulting in a significant impairment of the repair function of PDLSCs in the periodontal local tissue inflammatory environment.^31^^,^^32^ Therefore, exploring the potential mechanisms to improve the osteogenic differentiation ability of PDLSCs in the inflammatory environment and to restore the structure and function of damaged alveolar bone tissue is crucial for discovering new strategies for periodontal tissue regeneration therapy. In this study, to observe the effect of the proBDNF-SorCS2 signaling axis on the osteogenic differentiation ability of hPDLSCs, ALP, ARS staining, and Western blotting were used to observe the changes in the osteogenic differentiation ability of hPDLSCs. Our data have shown that blocking the proBDNF-SorCS2 axis by silencing SorCS2 can enhance early osteogenic activity (ALP) and mid-stage matrix protein production (OPN and RUNX2), as well as restore the expression of key markers of terminal osteoblast differentiation and mineral maturation (OCN). This confirms that in inflammatory conditions, the proBDNF-SorCS2 axis exerts a strong inhibitory effect on hPDLSCs throughout the osteogenic differentiation pathway, and inhibiting its expression can reverse this damage.

### Potential relationship between proBDNF-SorCS2 and the JNK signaling pathway

It was found that proBDNF and its receptor can activate the JNK pathway, a classical pathway by which proBDNF mediates cell death.^9^ In addition, Kaneko et al^33^ believed that the activation of the JNK signaling pathway could affect the osteogenic differentiation ability of hPDLSCs and the regeneration of periodontal tissues. Therefore, this study further explored whether the JNK signaling pathway was involved in the changes in inflammation level and osteogenic ability regulated by the proBDNF-SorCS2 axis in hPDLSCs. Consistent with the expression trend of the proBDNF-SorCS2 axis and inflammatory cytokines (IL-1β and IL-6), the expression of the JNK signaling pathway was also increased in the inflammatory state, and the expression level of the JNK signaling pathway was also significantly decreased after SorCS2 inhibition. Moreover, through pharmacologic activation approaches, we have found that the JNK pathway is a critical mediator of the proBDNF-SorCS2 axis. JNK activation reversed the beneficial effects of SorCS2 silencing, providing evidence that this pathway governs the inflammatory and osteogenic responses of hPDLSCs.

There are some limitations to our experiment. While our data provide in vitro evidence that the proBDNF-SorCS2 axis is a critical regulator of inflammation and osteogenesis in hPDLSCs, the therapeutic efficacy and safety of SorCS2 knockdown need to be evaluated in complex in vivo environments (eg, SorCS2 knockout mice). Promising strategies would include the local delivery of SorCS2-silencing agents (eg, small interfering RNA–loaded hydrogels) to periodontal defects to assess effects on inflammatory resolution and bone regeneration in vivo. Comprehensive preclinical toxicology studies (in periodontitis animal models) are also crucial for a comprehensive assessment of the safety and efficacy of SorCS2 inhibition. In our research, the acquisition of clinical samples is limited. Currently, only samples from patients at the end stage requiring surgery or extraction have been collected. Future studies using noninvasive methods (eg, monitoring proBDNF in gingival sulcus fluid) will clarify its dynamic changes in patients from health to illness. It is worth noting that this study focuses on the intrinsic signal transduction of hPDLSCs. The pathogenesis of periodontitis involves complex interactions among various cells. Our study specifically describes the role of the proBDNF-SorCS2 regulatory axis in hPDLSCs. Meanwhile, a recent study by Zhan et al^34^ highlights that macrophage senescence driven by glycolytic reprogramming constitutes another key cellular pathway that exacerbates the inflammatory microenvironment of periodontitis. In summary, these findings emphasize that periodontal inflammation is regulated by signal transduction mechanisms of different cell populations, suggesting that future multitargeted therapeutic strategies may be necessary. Therefore, future research should adopt coculture systems to determine how hPDLSCs affect the recruitment, polarisation, and function of macrophages, neutrophils, and T cells. Conversely, understanding how specific immune cells affect the activity of this axis within hPDLSCs will also provide a complete explanation for the cell interactions in periodontitis. Moreover, our study shows that LPS stimulation leads to an increase in the protein expression levels of proBDNF and its receptor SorCS2. However, whether LPS promotes gene expression at the transcriptional level or stabilizes proteins at the posttranslational level remains an unsolved mystery. Future research on this content will also be crucial for fully clarifying the initial steps of this pathway activation. For the exploration of potential downstream signaling pathways, our research has clearly demonstrated that the JNK signaling pathway is a key downstream effector factor of the proBDNF-SorCS2 axis. However, its potential interaction with other key inflammatory pathways (eg, the NF-κB signaling pathway) is also worthy of consideration.^35^, ^36^, ^37^ The NF-κB pathway is the main regulatory factor for inflammation in periodontitis. Moreover, it is known that NF-κB pathway interacts complexly with the JNK pathway.^38^ Therefore, the proBDNF-SorCS2 axis may indirectly regulate the activity of NF-κB by activating JNK. Clarifying the precise hierarchical relationship and potential synergistic effects between JNK and NF-κB in the signal transmission from the proBDNF-SorCS2 axis is an extremely attractive and important direction for future research.

**Fig. 6:**
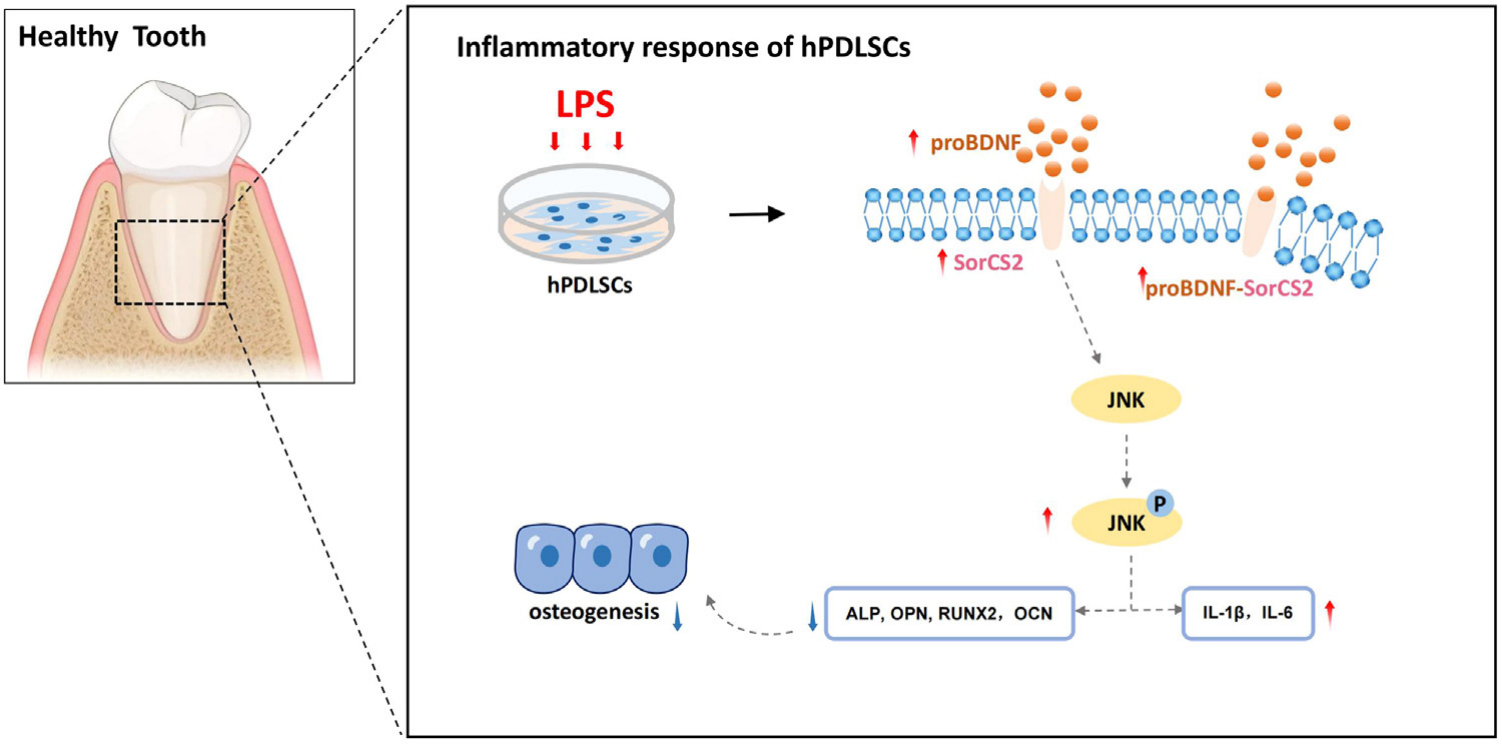
A schematic model of the proBDNF-SorCS2 signaling axis in hPDLSCs under inflammatory conditions. hPDLSCs were isolated from healthy tissues and cultured in vitro. LPS stimulation induced the expression of proBDNF and its receptor SorCS2. Their interaction activated the JNK signaling pathway (via phosphorylation), leading to increased inflammatory factors and suppressed osteogenic differentiation. BDNP, brain-derived neurotrophic factor; hPDLSC, human periodontal ligament stem cell; LPS, lipopolysaccharide; SorCS2, sortilin-related central nervous system expressed receptor 2.

In summary, our study reported the regulatory function of the proBDNF-SorSC2 signaling axis on PDLSCs under inflammatory conditions associated with periodontitis. Reducing SorCS2 expression could block the expression of related signaling pathways and further slow down the progression of the inflammatory response (Fig. 6).

## Author contributions

Conceptualisation: Pan, Jia, Guan, Hou; Methodology: Pan, Jia, Guan, Hou; Visualisation: Pan, Yang, Jia; Writing – original draft: Pan, Yang, Guan; Formal analysis: Yang; Data analysis: Zhao, Wang; Supervision, project administration, and funding acquisition: Hou.

## Conflict of interest

None disclosed.

## References

[1] Zhao X., Lin H., Ding T., Wang Y., Liu N., Shen Y. (2023). Overview of the main biological mechanisms linked to changes in periodontal ligament stem cells and the inflammatory microenvironment. J Zhejiang Univ Sci B.

[2] Wang L., Li X., Song Y. (2022). NELL1 augments osteogenesis and inhibits inflammation of human periodontal ligament stem cells induced by BMP9. J Periodontol.

[3] Xiang X., Hu Y., Song Z., Wang C. (2023). Knockdown of TRPM2 promotes osteogenic differentiation of human periodontal ligament stem cells by modulating NF-κB /NLRP3 pathway. Tissue Cell.

[4] Guan X., He Y., Li Y. (2022). Gremlin aggravates periodontitis via activation of the nuclear factor-kappa B signaling pathway. J Periodontol.

[5] Meng L., Yang P., Zhang W. (2023). Brain-derived neurotrophic factor promotes orthodontic tooth movement by alleviating periodontal ligament stem cell senescence. Cell Signal.

[6] Li X., Huang Y., Han Y., Yang Q., Zheng Y., Li W. (2022). LncPVT1 regulates osteogenic differentiation of human periodontal ligament cells via miR-10a-5p/brain-derived neurotrophic factor. J Periodontol.

[7] Kiyota M., Iwata T., Hasegawa N. (2024). Periodontal tissue regeneration with cementogenesis after application of brain-derived neurotrophic factor in 3-wall inflamed intra-bony defect. J Periodontal Res.

[8] Gibon J., Barker PA. (2017). Neurotrophins and proneurotrophins: focus on synaptic activity and plasticity in the brain. Neuroscientist.

[9] Li Q., Hu Y-Z, Gao S., Wang P-F, Hu Z-L, Dai R-P. (2023). ProBDNF and its receptors in immune-mediated inflammatory diseases: novel insights into the regulation of metabolism and mitochondria. Front Immunol.

[10] Leloup N., Chataigner L.M.P., Janssen BJC. (2018). Structural insights into SorCS2-nerve growth factor complex formation. Nat Commun.

[11] Glerup S., Olsen D., Vaegter C.B. (2014). SorCS2 regulates dopaminergic wiring and is processed into an apoptotic two-chain receptor in peripheral glia. Neuron.

[12] Vaegter C.B., Jansen P., Fjorback A.W. (2011). Sortilin associates with Trk receptors to enhance anterograde transport and neurotrophin signaling. Nat Neurosci.

[13] Glerup S., Nykjaer A., Vaegter CB. (2014). Sortilins in neurotrophic factor signaling. Handb Exp Pharmacol.

[14] Li Y., Guan X., He Y. (2023). ProBDNF signaling is involved in periodontitis-induced depression-like behavior in mouse hippocampus. Int Immunopharmacol.

[15] Janjić K., Agis H., Moritz A., Rausch-Fan X., Andrukhov O. (2022). Effects of collagen membranes and bone substitute differ in periodontal ligament cell microtissues and monolayers. J Periodontol.

[16] Guan X., He Y., Wei Z. (2021). Crosstalk between Wnt/β-catenin signaling and NF-κB signaling contributes to apical periodontitis. Int Immunopharmacol.

[17] Yang Y., Li M., Pan L. (2025). Inhibition of dynamin-related protein 1-dependent mitochondrial fission ameliorates apical periodontitis by attenuating NLRP3 inflammasome-mediated M1 macrophage polarisation. Int Dent J.

[18] Zhang L., Cheng L., Cui Y. (2021). The virulence factor GroEL directs the osteogenic and adipogenic differentiation of human periodontal ligament stem cells through the involvement of JNK/MAPK and NF-κB signaling. J Periodontol.

[19] Yu H., Wang P., Lu H. (2023). Effects of G-CSF on hPDLSC proliferation and osteogenic differentiation in the LPS-induced inflammatory microenvironment. BMC Oral Health.

[20] Lee K.M., Lee C.Y., Zhang G., Lyu A., Yue KKM. (2019). Methylglyoxal activates osteoclasts through JNK pathway leading to osteoporosis. Chem Biol Interact.

[21] Guo C., Yang X-G, Wang F., Ma X-Y. (2016). IL-1α induces apoptosis and inhibits the osteoblast differentiation of MC3T3-E1 cells through the JNK and p38 MAPK pathways. Int J Mol Med.

[22] Eraky S.M., El-Kashef D.H., El-Sherbiny M., Abo El-Magd NF. (2023). Naringenin mitigates thioacetamide-induced hepatic encephalopathy in rats: targeting the JNK/Bax/caspase-8 apoptotic pathway. Food Funct.

[23] Yang B., Wang L., Nie Y., Wei W., Xiong W. (2021). proBDNF expression induces apoptosis and inhibits synaptic regeneration by regulating the RhoA-JNK pathway in an in vitro post-stroke depression model. Transl Psychiatry.

[24] Iwasaki K., Peng Y., Kanda R., Umeda M., Ishikawa I. (2022). Stem cell transplantation and cell-free treatment for periodontal regeneration. Int J Mol Sci.

[25] Aby K., Antony R., Eichholz M., Srinivasan R., Li Y. (2021). Enhanced pro-BDNF-p75NTR pathway activity in denervated skeletal muscle. Life Sci.

[26] Aby K., Antony R., Li Y. (2023). ProBDNF upregulation in murine hind limb ischemia reperfusion injury: a driver of inflammation. Biology (Basel).

[27] Wang Y., Wang L., Sun T. (2023). Study of the inflammatory activating process in the early stage of Fusobacterium nucleatum infected PDLSCs. Int J Oral Sci.

[28] Kolar M.K., Itte V.N., Kingham P.J., Novikov L.N., Wiberg M., Kelk P. (2017). The neurotrophic effects of different human dental mesenchymal stem cells. Sci Rep.

[29] Glerup S., Bolcho U., Mølgaard S. (2016). SorCS2 is required for BDNF-dependent plasticity in the hippocampus. Mol Psychiatry.

[30] Yang F., You H., Mizui T. (2024). Inhibiting proBDNF to mature BDNF conversion leads to ASD-like phenotypes in vivo. Mol Psychiatry.

[31] Liu X., Tan G-R, Yu M. (2016). The effect of tumour necrosis factor-α on periodontal ligament stem cell differentiation and the related signaling pathways. Curr Stem Cell Res Ther.

[32] Cheng M., Zhou Q. (2020). Targeting EZH2 ameliorates the LPS-inhibited PDLSC osteogenesis via Wnt/β-catenin pathway. Cells Tissues Organs.

[33] Kaneko H., Hasegawa D., Itoyama T. (2022). Inhibition of c-Jun N-terminal kinase signaling promotes osteoblastic differentiation of periodontal ligament stem cells and induces regeneration of periodontal tissues. Arch Oral Biol.

[34] Zhan J., Kang J., Wei Y. (2025). Pexidartinib inhibits macrophage senescence through glycolysis in periodontitis microenvironment. Int Dent J.

[35] Zhou Z., Zhan C., Li W. (2025). Monocytic myeloid-derived suppressor cells contribute to the exacerbation of bone destruction in periodontitis. J Transl Med.

[36] Li X., Liu X., Zhou J., Zhang P., Chen S., Bai D. (2025). Human dental follicle stem cell-derived exosomes reduce root resorption by inhibiting periodontal ligament cell pyroptosis. Stem Cell Res Ther.

[37] Chen L., Liu Y., Yu C. (2025). Induced pluripotent stem cell-derived mesenchymal stem cells (iMSCs) inhibit M1 macrophage polarization and reduce alveolar bone loss associated with periodontitis. Stem Cell Res Ther.

[38] Zhao S-Y, Zhao H-H, Wang B-H, Shao C., Pan W-J, Li S-M. (2023). Rhein alleviates advanced glycation end products (AGEs)-induced inflammatory injury of diabetic cardiomyopathy in vitro and in vivo models. J Nat Med.

